# Updated analysis of the Bronze Age population genetics from Bezdanjača Cave – the case of I2a1a male individuals

**DOI:** 10.3325/cmj.2026.67.247

**Published:** 2026-06

**Authors:** Damir Marjanović, Jelena Šarac, Dubravka Havaš Auguštin, Ivor Janković, Siniša Radović, Dragan Primorac, Mario Novak

**Affiliations:** 1 Centre for Applied Bioanthropology, Institute for Anthropological Research, Zagreb, Croatia; 2 Department of Genetics and Bioengineering, International Burch University, Sarajevo, Bosnia and Herzegovina; 3 Faculty of Biotechnology and Drug Development, University of Rijeka, Rijeka, Croatia; 4 Institute for Quaternary Paleontology and Geology, Croatian Academy of Sciences and Arts, Zagreb, Croatia; 5 St. Catherine Specialty Hospital, Zagreb, Croatia; 6 Faculty of Dental Medicine and Health, Josip Juraj Strossmayer University of Osijek, Osijek, Croatia; 7 School of Medicine, Josip Juraj Strossmayer University of Osijek, Osijek, Croatia; 8 Medical School, University of Split, Split, Croatia; 9 Department of Biochemistry & Molecular Biology, The Pennsylvania State University, State College, PA, USA; 10 The Henry C. Lee College of Criminal Justice and Forensic Sciences, University of New Haven, West Haven, CT, USA; 11 Regiomed Kliniken, Coburg, Germany; 12 Medical School, University of Rijeka, Rijeka, Croatia; 13 Gandhinagar Campus, National Forensic Sciences University, Gandhinagar, India; 14 Department of Archaeology and Heritage, Faculty of Humanities, University of Primorska, Koper, Slovenia

## Abstract

This case report presents an updated interpretation of genetic and chronological data from human remains discovered in Bezdanjača Cave, a Bronze Age burial site located in the Lika region of Croatia. The cave contains a complex necropolis with at least 57 graves and up to 200 individuals, which indicates its use as a collective burial site during the Middle and Late Bronze Age. Based on ancient DNA analysis, 13 males were identified among 38 analyzed individuals, with the majority belonging to the Y-chromosome haplogroup R1b, commonly associated with Bronze Age populations. However, two individuals were assigned to haplogroup I2a1a (I-Y3120). The I2a lineage has deep roots in Europe, and its presence has been confirmed in prehistoric contexts in Croatia and the region. However, newly obtained radiocarbon dates from occipital bones reveal that at least one of the two I2a1a individuals from Bezdanjača Cave dates to the Early Modern period (1645-1950 calibrated CE), which indicates that the remains were deposited in the cave much later than previously assumed. At the same time, these new data do not contradict the possible presence of the I2a1a lineage in Bronze Age populations in this area, as Bronze Age I2a1a samples have been reported from other archaeological sites in Croatia and the wider region. These findings, presented here for the first time, highlight the risks of assuming chronological homogeneity based solely on archaeological context and demonstrate the necessity of direct radiocarbon dating when integrating archaeological and genetic data. An interdisciplinary approach and careful chronological verification in ancient DNA research are essential to avoid misinterpretations in broader population genetic studies.

Bezdanjača Cave is located on the Vatinovac hill in the mountains of the Lika region in Croatia. The cave was discovered in 1960, and the site was explored in 1965 (1). The entrance to the cave is formed as a vertical shaft, from which the site derives its name – the word “*bezdan*” in the Croatian language means “abyss” or “bottomless pit.” The toponym Bezdanjača thus reflects both the morphology of the site and its striking visual impression within the karst landscape of the Lika region (1). The cave is difficult to approach, so it may have been undisturbed for thousands of years until its discovery (2). It is complex and consists of multiple vertical and horizontal channels with a total length of 1176 m and a depth of 200 m (3). The archaeological artifacts, as well as human and faunal remains, discovered during the research showed very interesting results. Namely, the cave seems to have been used as a burial site during the Bronze Age, with the cave itself representing a kind of tomb. Human remains were laid on the cave floor or sometimes in niches along the cave walls without inhumation or covering the skeletons (Figure 1).

**Figure 1:**
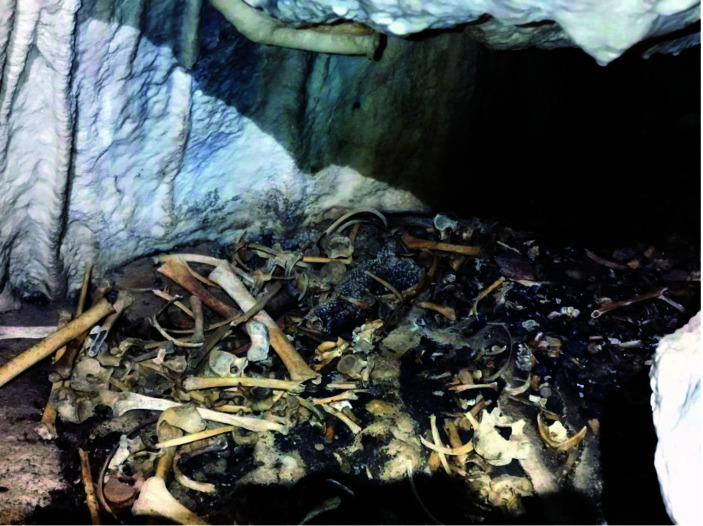
Skeletal remains noted in the Bezdanjača Cave during a visit in 2023.

Within the cave, 57 graves were identified with up to 200 individuals. Ten graves contained more than one deceased (five to 20 individuals) (1). A short horizontal shaft west of the entrance could also have been used for habitation by the living, while the other parts of the cave served as a burial site (4). Traces of burning, structures made from wood and stone, burnt animal bones, ceramic finds, ochre, burnt sticks, wooden spoons, and other items indicate complex burial rituals (1,3). Furthermore, complete or partial animal skeletons were laid alongside a portion of human skeletons (1,3,5). The chronological estimation of the cave's use had been assessed via the horizontal stratigraphical position of the graves, along with pottery typology. Based on the dating, the necropolis was divided into two segments: the earlier (Middle Bronze Age) and later (Late Bronze Age) phase (1). Several direct radiocarbon dates of human remains (mostly long bones and wooden artifacts) place the use of the cave in the second half of the second millennium BCE (6-8).

Recently, human remains from Bezdanjača gained renewed scientific attention with several studies conducted, including conventional bioarchaeological analysis (9,10) as well as carbon and nitrogen isotopic analyses (11,12). Furthermore, ancient DNA (aDNA) analysis was performed on 38 individuals from the cave, and these samples were included in several large-scale genomic studies as representatives of Bronze Age populations (6,13-15). The aim of this study was to present an updated interpretation of genetic and chronological data from human remains discovered in Bezdanjača Cave, based on new radiocarbon dating of three skulls.

## Case report

This study presents new direct radiocarbon dating of occipital bones from three Bezdanjača skulls. Two align very well with the already known accelerator mass spectrometry (AMS) dates (BzV-18a, UCIAMS233512, 1422-1281 cal BCE, 3090 ± 25 before present [BP]; BzV-28a, UCIAMS233513, 1422-1281 cal BCE, 3090 ± 25 BP) confirming the Bronze Age chronology of the cave. However, one date is much more recent (BzV-11a, UCIAMS233511, 1645-1950 cal CE, 215 ± 20 BP), placing this cranium to the Early Modern period.

**Figure 2:**
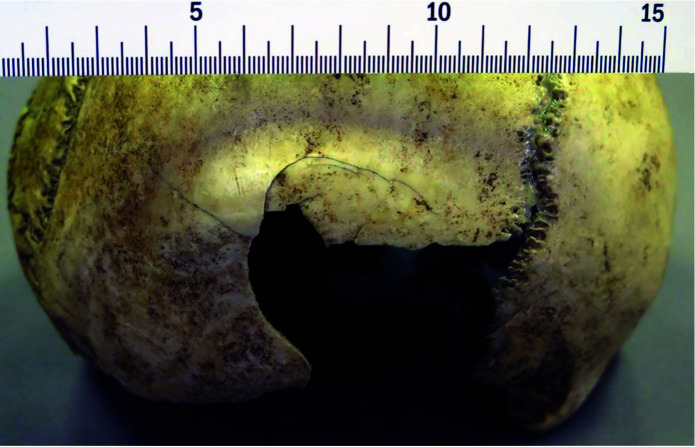
Skull Bzv-11a with a massive perimortem injury to the right parietal bone.

**Figure 3:**
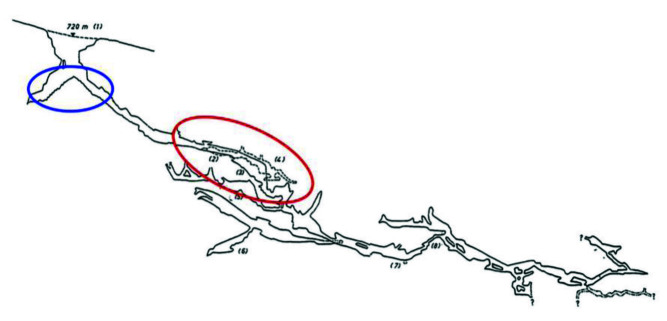
A plan of Bezdanjača Cave: blue ellipse – recent human and faunal remains; red ellipse – Bronze Age human and faunal remains and archaeological artifacts (taken from (5), with permission from the publisher, adjusted by M. Novak and J. Šarac).

**Figure 4:**
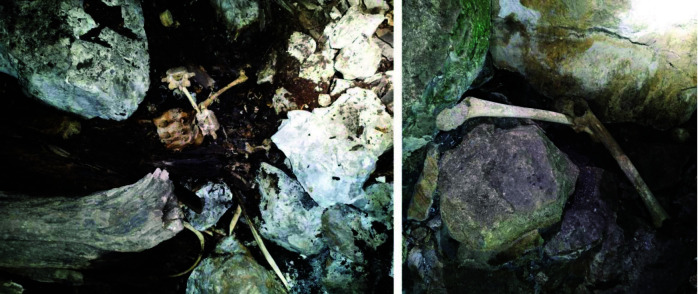
Human skeletal remains found at the cave entrance during a visit in 2023.

The skull BZV-11a exhibits a massive blunt-force perimortem injury to the right parietal bone, a clear sign of intentional violence (Figure 2). It belonged to an adult male whose remains were deposited in the cave sometime between the mid-17th and the mid-20th centuries CE, as confirmed through direct radiocarbon dating. The partially preserved skull (only the bones of the left side are present) of BzV-10a, belonging to an adult male, also probably comes from a similar context. The field diaries and excavation documentation do not specify that any of the remains could be dated to recent periods. However, Malinar (16) mentioned the find of two human skeletons that most likely ended up in Bezdanjača during World War II, as well as skeletal remains of animals that accidentally fell in, located at the bottom of the shaft, right at the entrance of the cave (Figure 3). This was confirmed by a speleological visit to the cave in October 2023, conducted by a research team that included the co-authors of this paper. During this visit, adult human remains were found directly at the bottom of the shaft, together with a large quantity of faunal remains (17). This finding is reported here for the first time (Figure 4). Based on all this, the skulls BzV-10a and BzV-11a might belong to these individuals. However, direct association of the remains cannot be made with certainty.

Fortunately, a large majority of human skeletal remains were found along archaeological finds from the Bronze Age and were located deeper in the horizontal channels and caverns of the cave (1) (Figure 3).

The results of uniparental markers, especially the Y chromosome haplogroup analysis of the tested Bezdanjača samples also yielded intriguing results. Namely, among 38 analyzed samples from the cave, 13 males were detected. The vast majority of them (11 samples; >84%) could be attributed to the R1b haplogroup, the most prevalent haplogroup among Bronze Age populations and associated with Caucasus/steppe ancestry (18). Given the high frequency of R1b, a shared paternal lineage might initially be suspected. However, kinship analysis demonstrated that, although nine samples were confirmed relatives belonging to four families, only two R1b individuals were present within these family groupings. This indicates that the nine R1b males are not closely related to one another and do not belong to a single paternal lineage.

The other two male samples (BzV-10a and BzV-11a) were assigned to the I2a1a (I-Y3120) haplogroup, based on ISOGG v. 15 (6,13). BzV-11a, the sample that was recently carbon-dated to a much younger historic period carried the I2a1a haplogroup. It is probable that the skull BzV-10a is also more recent and belongs to one of the two individuals found at the entrance to the cave, which would link the genetic and archaeological data into a common context. However, until the skull BzV-10a is AMS-dated this still remains a hypothesis.

## Discussion

When looking more closely at the I2a1a haplogroup, ancient DNA studies have shown that haplogroup I (including early I2 lineages) was common among Mesolithic foragers across Europe (19-21). However, in prehistoric Croatia, I2a1a appears to have been sporadically present (6,15,22,23). It has also been detected in two samples from a 6200-year-old mass grave in Potočani, Croatia (24) and in three unpublished prehistoric Croatian samples (David Reich, personal communication). This suggests its long-term presence in this region.

The two I2a1a individuals from the Bezdanjača Cave that were initially attributed to the Bronze Age primarily on archaeological and contextual grounds, appear to be chronologically from the Early Modern period. However, until recently this information was not available and, due to the lack of this data, many ancient DNA studies in the last decade have included these samples as Bronze Age samples in their analysis.

This finding highlights the complex history of the cave and points to the risk of assuming chronological homogeneity based only on contextual association. The presence of skeletal remains linked to the Early Modern period in the Bezdanjača Cave demonstrates the value of direct dating of all skeletal material when interpreting archaeological finds and drawing firm conclusions. Bioarchaeological interpretations must be made with caution, ensuring that human remains assigned to a certain time period are securely dated rather than placed chronologically based only on spatial proximity or associated artifacts.

However, these new data do not contradict the possible presence of the I2a1a lineage in Bronze Age populations in this area. All SE European “Bronze Age I2a1a samples” currently reported in the literature are listed in Table 1, once again proving the presence of this Y haplogroup in this area much before medieval migrations.

**Table 1:** Ancient I2a1a samples from Southeast Europe dated to the prehistoric period. The samples in the focus of this study are marked with an asterisk

Genetic ID	Skeletal ID	Age (cal = radiocarbon dating)	Locality	Country	Reference
I10062.AG	P8L12 (mass burial)	4300-3900 BCE	Potočani; Lasinja culture (Požega-Slavonia County)	Croatia	Novak et al 2021 (24)
I10072.AG	P8L3-3 (mass burial)	4300-3900 BCE	Potočani; Lasinja culture (Požega-Slavonia County)	Croatia	Novak et al 2021 (24)
I15621	1737; 25	4500-3500 BCE	Urziceni	Romania	Lazaridis et al 2022 (6)
I17916	3562; M120	2100-1800 BCE	North Banat District, Kikinda, Mokrin Necropolis	Serbia	Lazaridis et al 2022 (6)
I7137	URZI75; gr. 75	4157-3966 cal BCE (5215 ± 25 BP, PSUAMS-4230)	Urziceni	Romania	Lazaridis et al 2022 (6)
I19454	Tell Kran 2009; Burial 8, spit 5, sample 35	3000-2000 BCE	Kazanlak, Tell Kran (Central, Yasenovo)	Bulgaria	Lazaridis et al 2022 (6)
I10479	CARM_33B; Grave 33B	2150-1850 BCE	Cârlomănești Arman	Romania	Lazaridis et al 2022 (6)
YUN037	Burial 8 Ind 1	first sibling of YUN038, the dates are the same	Pazardzhik	Bulgaria	Penske et al 2023 (25)
YUN038	Burial 8 Ind 2	2911-2765 cal BCE (4248 ± 24 BP, MAMS-45494)	Pazardzhik	Bulgaria	Penske et al 2023 (25)
PIE078	P16 N513	3335-3026 cal BCE (4463 ± 25 BP, MAMS-47846)	Giurgiu County	Romania	Penske et al 2023 (25)
I18721*	3781; BzV 11a	1500-1000 BCE, corrected to 1645-1950 cal CE, 215 ± 20 BP	Bezdanjača Cave	Croatia	Lazaridis et al 2022 (6)
I18719*	P3779; BzV 10a	1500-1000 BCE?, most probably needs correction	Bezdanjača Cave	Croatia	Patterson et al 2022 (13)
I43580.TW	P15279 (G6)	4700-4500 BCE	Kotlina (Osijek-Baranja County)	Croatia	unpublished data (Reich, personal communication)
I42882.TW	P15108 (SJ 203)	2200-2100 BCE	Zeleno Polje (Osijek-Baranja County)	Croatia	unpublished data (Reich, personal communication)
I40070.TW	P14363 (G5)	6200-5300 BCE	Vinkovci (Vukovar-Syrmia County)	Croatia	unpublished data (Reich, personal communication)

Ancient DNA and population genetic studies differ in certain important aspects. Namely, ancient DNA studies rely on small, preservation-dependent samples from specific archaeological contexts, providing temporally precise but statistically limited “snapshots” of past populations. In such small data sets, lineages present at moderate frequencies may be missed, and burial assemblages may represent kin-based or socially stratified groups rather than a wider population. In contrast, modern population-genetic surveys are typically large, structured, and geographically representative, but lack a temporal perspective. Therefore, integrating evidence from both ancient DNA and modern population-genetic studies provides the most comprehensive framework for reconstructing past population structure and genetic history. It is also imperative to generally broaden the sample size for population analysis of ancient DNA from SE Europe to its maximum extent. There is a need for deeper ancient DNA analysis of Copper and Iron Age sites, including a wider temporal context (eg, Late Middle Ages), extensive population-scale ancient DNA studies and direct radiocarbon dating of samples, as well as their interpretation in an interdisciplinary population genetic framework.
